# Complications and Patient Satisfaction After Endoscopic Radial Artery Harvest: A Retrospective Cohort Study

**DOI:** 10.3390/jcm15093338

**Published:** 2026-04-27

**Authors:** Christian L. Carranza, Louise Uth, Janus Christian Jakobsen

**Affiliations:** 1 Department of Cardio-Thoracic Surgery, The Heart Centre, Rigshospitalet, Copenhagen University Hospital, 2100 Copenhagen, Denmark; 2 Copenhagen Trial Unit, Centre for Clinical Intervention Research, Copenhagen University Hospital, Rigshospitalet, 2100 Copenhagen, The Capital Region, Denmark; 3 Department of Regional Health Research, The Faculty of Health Sciences, University of Southern Denmark, 5230 Odense, Denmark

**Keywords:** endoscopic surgery, radial artery, vessel harvest, ERAH, EVH, MICS, CABG

## Abstract

**Background/Objectives**: This study aimed to assess complications after endoscopic radial artery harvest, evaluate patient satisfaction, and assess the feasibility of a questionnaire interview. The primary aim was to assess neurological damage after endoscopic radial artery harvest (ERAH), and the secondary aim was to assess the feasibility of a newly constructed questionnaire in Danish. **Methods**: From June 2010 through October 2012, 108 patients underwent endoscopic radial artery harvest for coronary artery bypass grafting (CABG) at our institution. A total of 100 patients were interviewed by phone between one and two years after the operation. The interviews included questions about infection, bleeding, neurological damage, vascular perfusion of the hand, re-intervention, and general satisfaction with the procedure. **Results**: The described cohort was mainly male (88.9% 95% confidence interval (CI) 90.5–98.4%) with a mean age of 60.8 years (standard deviation (SD) 9.0). The self-reported incidence of postoperative antibiotic treatment was 7.0% (95% CI 3.1–14.4%), sensory deficit 18.0% (95% CI 11.3–27.2%), pain 8.0% (95% CI 3.8–15.6%), motoric deficit 4.0% (95% CI 1.3–10.5%) and cold or pale fingers 9.0% (95% CI 4.5–16.8%). At a follow-up time with a mean of 1.40 years (range 0.97–2.37 years, SD 0.36), all incisions were healed satisfactorily, 12.0% (95% CI 6.6–20.4%) reported sensory deficit, 7.0% (95% CI 3.1–14.4%) reported pain, 2.0% (95% CI 0.3–7.7%) reported motoric deficit, and none had a tendency towards cold fingers. The mean duration of antibiotic treatment was 8.7 days ± 2.3. When asked to rate the endoscopic procedure points of satisfaction from 0 (worst) to 10 (best), the mean was 9.7 ± 0.7. **Conclusions**: This study reports the occurrence of surgical complications immediately after ERAH, with an occurrence of self-reported neurological deficits of 18%. A questionnaire was feasible in a cohort of postoperative patients receiving radial artery harvest.

## 1. Introduction

Coronary artery bypass grafting (CABG), also named bypass surgery, has, for more than 40 years, shown the importance of coronary artery revascularization in patients with arteriosclerosis. CABG enhances quality of life by removing chest pain (angina pectoris) as well as prolongs life expectancy in these patients [1].

Realizing the importance of low operative risk and high long-term patency in CABG procedures has been an important research area for many years. Superior long-term survival in patients receiving arterial grafting using the left internal mammarian artery (LIMA) was described in the mid-1990s [2] and it has been perceived as the primary graft choice since the 1970s. The secondary graft choice has been debated. Initially, there was a high usage of saphenous vein grafts (SVG), and it is still many clinics’ second choice, but in 1971, the radial artery was first used by Carpentier. It has shown superior outcomes, though evidence remains limited to a few clinical randomized trials [3]. Nevertheless, European guidelines recommend using arterial grafts in young people or patients with long life expectancy, where long-term survival benefits have the greatest impact [4]. This is also supported by the newest international recommendations of conduit choice [1,5,6]

To reduce operative risk, graft harvesting techniques have also undergone technical evolution. Classic open graft vessel harvesting techniques, including bridging, were replaced during the period 2000–2005 by endoscopic harvesting techniques, which are now the primary choice of technique in some parts of the world [7]. The radial artery (RA) can be harvested with an open technique (ORAH) [8] or an endoscopic technique (ERAH) [9]. This shift in techniques, seen in vein graft harvesting, was later introduced in radial artery graft harvesting [10]. A total of six randomized clinical trials have tried to show the benefits and disadvantages of ERAH versus ORAH [11]. Most importantly, the conclusions in general were that ERAH is a safe procedure with reduced postoperative complications compared with ORAH [12]. Even though there is slowly growing evidence towards ERAH, no studies were found to have focused on an interview of the patients as opposed to ORAH [13]. Besides quantifying self-reported complications, this study was used as a precursor to the randomized “Neurological deficits in Endoscopic versus Open radial artery harvest” (NEO) trial [14], where a modification of the RAPCO interview will be used [15], as well as the questionnaire explored in this study, if found feasible.

## 2. Materials and Methods

This is a retrospective cohort study of consecutive patients after ERAH at Gentofte Hospital and Rigshospitalet in Copenhagen, Denmark. Inclusion criteria were adult patients (>18 years) with multivessel disease admitted for bypass surgery at the named institutions and operated on from June 2010 until October 2012, in whom the primary surgeon planned the use of the radial artery as a graft. Exclusion criteria were non-eligibility for radial artery harvest assessed by a modified Allens test [16], availability of surgical staff able to perform ERAH and patients not able to comply with postoperative medication of Amlodipine for 3 months. Study size was defined by the timeframe of inclusion.

### 2.1. The Procedure

**Figure 1:**
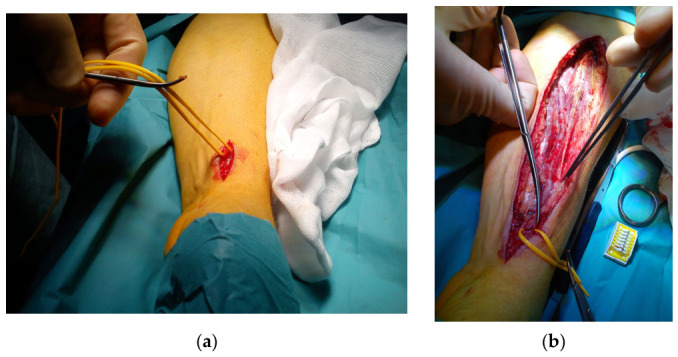
Endoscopic versus open harvest of the radial artery: (**a**) endoscopic radial artery harvest (ERAH), (**b**) open radial artery harvest (ORAH).

Surgical technique for endoscopic graft harvest used was standard technique using Vasoview Hemopro 2 (manufactured by Maquet, Getinge) [17]. Non-dominant arm was preferred. The day before surgery, a modified Allens test [16] was performed. If re-perfusion of the palm by the ulnary artery was achieved in less than 10 s, the patient was deemed suitable for radial artery harvest (positive test). In cases of a negative test, the patient was revascularized using other grafts, alternatively using the dominant arm. During surgery an incision of about 2 cm. close to the wrist was done just overlaying the RA. The artery was dissected free, and a small vessel clamp is positioned over the artery. A pulse oximetry on the same hand’s second finger was used to re-do the Allen test by directly clamping the artery and observing the pulse curve. If the saturation was unchanged and the pulsatile measurements were satisfying, the procedure was continued after giving 5000 units of heparin. Through the 2 cm incision, a space to fit the endoscopic port was dissected. A straight endoscope mounted with a dissection tip was used to free the RA pedicle, including the veins. Side branches were ligated using the Hemopro 2 device, and when complete, the proximal part was divided through a stab incision at the elbow bend. Finally, the RA pedicle was extracted at the wrist and divided. Stumps were ligated, incisions were sutured, and the arm was bandaged. Both ERAH and ORAH are shown in Figure 1.

The RA was most often used as a composite graft with a proximal anastomosis to the LIMA (also named RA-LIMA Y anastomosis). The revascularization technique followed normal department protocol. All patients were operated using extracorporeal circulation (heart-and-lung machine; ECC) and cardiac arrest (CABG) or beating heart—also named off-pump cardiac bypass grafting (OPCAB). The use of the right internal mammary artery (RIMA) was also permitted, as some patients had both mammary arteries used. Accordingly, study patients were not treated in any way other than the normal procedure at the department. Also, with respect to RA harvest, it had been harvested using the open classical technique for many years [8] and using it as much as possible was the departmental procedure.

Likewise, the postoperative routines did not differ from department standards. All patients were treated with a calcium-channel blocker, Amlodipine 10 mg, starting the day before surgery and continuing for 3 months postoperatively to prevent arterial graft spasm.

### 2.2. Data Were Collected as Follows

- Datasheet containing demographic data, pre-operative data, postoperative data and data about complications (Appendix A).
- A Danish questionnaire asking the patient about infection, hematoma, pain/discomfort, paresis, limb coldness and angina recurrence. Satisfaction with the minimal invasive procedure and an economic appraisal were included (Appendix B).

Two main predefined outcomes were specified. First was the assessment of neurological deficits following ERAH, used for power calculations planning a randomized clinical trial [14]. Second was the evaluation of whether a telephone interview questionnaire would be feasible for use in the same later randomized clinical trial [14].

A study nurse collected data sheets using the electronic patient journal OPUS (CSC Scandihealth). Any supplemental data needed for the datasheets was found in the Dendrite Clinical Systems “Patient Advocate Tracking System” (PATS). Datasheets were filled in near relation to and needed to be completed before the telephone interview was executed. Data were searched using the patient’s unique identifier (Social Security number) in the electronic medical record available in the Capital Region of Copenhagen.

The questionnaire used was newly constructed by the main author for the purpose of this study and as a possible addition to the NEO Trial, if it was assessed as usable.

Questionnaire data were collected by telephone interview. A study nurse performed the interviews and answers were noted on a clinical research form. Between one and two years after the procedure, a study nurse would perform the telephone interview. The interviews were conducted by contacting individual participants, and a predefined interview protocol (Appendix B) was followed systematically.

Of the 108 patients, 6 died before follow-up. One patient did not want to participate in the telephone interview, one patient was lost to follow-up due to missing contact information and one patient ended up with the RA harvested by ERAH, but without bypass operation performed. The reason this patient did not receive a bypass procedure was pericardial adhesions, making open heart surgery impossible. This patient’s postoperative admission data were not relevant, though the interview data on postoperative limb complications remain relevant and should be included in the study. In total 100 telephone interviews were completed, and 107 postoperative patient data could be included in the analysis.

Data were summarized and statistically processed using Excel (Microsoft). Categorial variables were presented as frequencies and percentages. Wilson confidence intervals (CIs) were calculated. Continuous variables were presented as means as standard deviation (SD) was calculated.

The study was approved by the Danish Ethics Committee by 20 February 2012 (application number: H-4-2012-FSP 16). The study was approved by the Danish Data Protection Agency by 28 August 2012 (approval number: 2007-58-0015). Patients provided a general written consent the day before the operation. Patients provided verbal consent to this exact study at follow-up.

The study conformed to The STROBE guidelines [18].

## 3. Results

Cohort demographics can be seen in Table 1. The patients had a mean age of 60.8 years and were mainly male (88.9%). Patients were comparable to the standard bypass patient population in Denmark. There was a presence of slight overweight, with a BMI mean and median of around 28. Likewise, most patients, by far, were suffering from hypertension and hypercholesterolemia. A third of patients were active smokers, and a third were suffering from diabetes. Few had formerly had a cerebral stroke as well as chronic obstructive pulmonary disease, peripheral neuropathy, preoperative dialysis and preoperative nephropathy.

As described in Table 1, there was an overweight of patients with left main stem stenosis, and we also included subacute patients, but no acute cases. No patients had one-vessel disease; these are seldom referred for surgery and would not be included, as they would not need their RA vessel harvested. Besides these two exclusion criteria of acute patients and one-vessel disease the population represented all come with referrals to our department with an RA suitable for graft use. A subacute patient would be one operated on during the same hospitalization as the primary admission, whereas an acute patient would be one operated within the first 24 h of admission.

EuroSCORE II was used to risk score patient mortality risk within 30 days [19]. Patient risk score was, in general, low, with a EuroSCORE II median of 1.14% corresponding with primary elective cases.

The perioperative data (Table 2) show that the majority of operations performed were single-procedure interventions (bypass surgery alone) and were mainly performed using extracorporeal circulation (ECC, i.e., a heart-lung machine). These were classified as coronary artery bypass graft surgery (CABG) with the use of ECC, as opposed to off-pump coronary artery bypass surgery (OPCAB). When the surgical procedure also included replacement of the aortic valve, this was named aortic valve replacement (AVR), and when pulmonary vein ablation was performed due to atrial fibrillation, this was noted in four and five cases, respectively. Only one procedure was noted as “other” because this patient did not receive coronary artery bypass due to pericardial adherence. In contrast, the arterial artery still had been harvested before the bypass procedure was abandoned.

Most patients received ≥3 grafts (i.e., 82.2%) and only one proximal anastomosis (i.e., 83.8%), reflecting the on-location high rate utilizing Y-anastomosis (RIMA or RA) to LIMA technique for multi-vessel CABG. Most used was the RA-LIMA Y-anastomosis, performed in 75% of patients. Vein grafts were only used in 14.8% of patients. This reflected a clinic where most patients received total arterial revascularization. Only one patient received no arterial mammarian grafts, which was the same patient who did not receive coronary artery bypass surgery due to pericardial adherence.

Postoperative general and arm complications are listed in Table 3. One patient had no registration of general complications but only arm complications. As seen, general complications, such as atrial fibrillation (23.4%), were expected, consistent with other literature reporting 22–37% [20,21]. Likewise, reoperation due to bleeding (4.7%) did not deviate from the literature [22], but early graft failure and re-operation CABG (3.7%) was somewhat higher than the literature, but still, the total number of occurrences was few and thereby not necessarily a significant difference [23]. Mediastinitis occurrence (3.7%), cerebral stroke post-surgery (2.8%), and nephrological complications (4.6%) also equaled that in the literature [24,25,26]. Finally, postoperative mortality within 30 days of 1.9% was slightly higher than predicted by the mean EuroSCORE II (Table 1) of 1.52%, but with a mortality of only two persons, this is by far a non-significant deviation.

The occurrence of post-procedure arm complications when using ERAH is very low, as seen in Table 3.

Using the interview scheme (Appendix B), we found very few patients assessed complaints after ERAH (Table 4). Most significantly, patients reported sensibility disturbances in the arm with an 18.0% occurrence after the operation, dropping by 33% to 12.0% by the interview follow-up. Most patients reported it to involve the thumb (44.4%), but nearly as many answered “other”, thereby indicating wrist (11.1%), hand (5.6%), arm (5.6%), or scar (16.7%). Half of patients had described a “pricky” sensation, while the rest described decreased sensibility (22.2%) and sleeping sensation (16.7%), but only one patient (5.6%) with ceased sensibility.

When asked about pain, only 8% experienced pain after the operation. Most described a location of “other” than the fingers, mainly localized to the scar and arm. After one year, 7% still had pain, mostly described as neurological pain. Even the ones who had answered “other”, 50%, were mainly describing a multitude of neurological pain deficits also located to the rest of the arm.

Few patients (4%) had power reduction, which was reduced to 2% at the time of the interview. Of the four patients, one had a non-significant power reduction, two had a reduction in some movement, and the last, described as “other,” was, in reality, a patient-perceived tiredness in the hand.

The shortest time point was 0.97 years and longest 2.37 years with a mean of 1.4 years and standard deviation 0.36 years.

In summary the results from this ERAH population showed self-reported occurrence of sensory deficit 12%, strength deficit 2%, pain 7%, and cold fingers 8% one-year after surgery.

## 4. Discussion

We sought to describe patients’ perceived consequences after ERAH to better plan the NEO Trial, including a more accurate power calculation. These results describe a cohort of the first 108 procedures performed. Therefore, this study might be influenced by learning-curve issues, such as a higher conversion rate to open harvesting, larger incisions, and a higher risk of neurologic damage and hematoma formation. Fortunately, we had a loss to follow-up of only eight participants (7.4%), of whom two (1.8%) were due to missing contact information at follow-up or refusal to participate, and six (5.6%) were due to death before follow-up. This survivorship bias and non-response bias limit generalizability, but are of a low percentage, probably not affecting data significantly.

Our participant population was representative of all-comer-referred patients for bypass surgery during the specified time period, but no acute patients were included. This meant that participants were, in general, low risk patients as shown by the EuroSCORE II. Further elaboration on the physical impairment of the patients could have been shown using the American Society of Anaesthesiologists (ASA) grading, but due to this being non-cardiac surgery specific, the EuroSCORE II was preferred [19,27].

The RAPCO randomized clinical trial compared RA grafting with the right internal mammary artery (RIMA) or venous grafting [28]. We decided to use their hand function questionnaire for the NEO Trial since it seemed to be the most complete. The RAPCO group managed to conduct up to 14 years of follow-up with 408 patients who answered the questionnaire [15,29]. This questionnaire included seven statements about daily life symptoms or limitations of the hands and forearms, as well as four questions concerning arm or leg scars. All patients in RAPCO were ORAH. We would anticipate an impact on quality of life with ERAH compared with ORAH, as several studies have shown fewer neurological deficits and greater patient satisfaction after ORAH [30,31,32,33].

In our population of ERAH patients, we needed a preliminary interview structure to quantify other, more general postoperative complications. If we had used the RAPCO questionnaire in our ERAH implementation population, we probably would get a very low prevalence of negative outcomes. We therefore concentrated our efforts on designing a new telephone interview questionnaire to quantify general postoperative complications and qualifying this new questionnaire to supplement the RAPCO questionnaire, when both were to be used in the NEO Trial. Since the questionnaire was newly constructed, there was no means to assess validity or reliability before the study.

Our interview study is biased by the time passed. Because we ask about things concerning hospitalization one-year post-surgery, this will be susceptible to recall bias. This was noticeable by the fact that six participants were unable to state which donor arm was used. On the other hand, any patients who still had trouble with their arm after one year would probably be very reliable in recalling whether problems arose just after the operation.

Non-blinded interviewers may bias this study. In order to minimize this bias, most interviews were conducted by a study nurse with no relation to the endoscopic intervention. A surgeon and another available nurse did the rest.

So far, six randomized clinical trials have sought to demonstrate differences between ORAH and ERAH. The conclusion was a reduced incidence of postoperative wound infection and wound pain, improved patient satisfaction, and cosmetic results in one study [34]. Two studies showed better or the same preservation of endothelial integrity [35,36], and another two studies showed no difference between ORAH and ERAH [37,38]. The last study interviewed half as many patients as our study, but supported the same findings of high self-reported patient satisfaction and low postoperative complication rates, concluding that ORAH was safe with respect to graft quality and arm function [30]. The largest study included 200 patients, two studies included 50 patients, and the other studies included 119, 60, and 54, respectively. Very recently, a seventh randomized trial (NEO Trial) was published, building on the experience from this study for power calculations [39].

In this study, we asked participants about infections occurring after ERAH. This could be investigated in depth in future trials using biomarkers of inflammation or infection. Likewise, a further perspective of cold reactive protein activation in cardiac surgery could be explored, though in many departments, as described in this paper, the ECC is run in normothermia. Even though this is normal mainstream practice, there could be a risk for activation to occur.

Participants included in this study all had at least one arterial graft. Primarily used is the LIMA and, secondarily, the RA and/or the RIMA. Arterial grafts are chosen for younger patients or when clinics have a historical preference for this practice. Many still use the SVG as a secondary graft, but guidelines advocate the use of LIMA and arterial grafts in certain patients [1,5,6]. At Gentofte Hospital, the use of RA as a secondary choice of graft was customary, but at Rigshospitalet, it was only preferred for younger patients. This mixed procedure is also reflected in the study population being somewhat younger than average. Percutaneous intervention is a possibility as a treatment of ischemic heart disease, but all study patients were discussed at a Heart Team Conference and referred for open cardiac surgery. Over the last years, the indication for percutaneous versus open surgery has shifted. Especially since the SYNTAX trial, many considerations of when to choose which strategy have been described [40,41]. Due to the time elapsed since this study’s inclusion period, the general population has probably changed demographically, and thus the results cannot be generalized to the current bypass population.

The purpose of this trial was never to compare interventions; hence, there is no control group of open-harvested participants, which would have made the paper more all-round descriptive of patients with radial artery harvest. The generalizability of this study is unfortunately low due to the bias presented.

No sample size calculations were performed when planning the paper, since no control cohort was planned. We would, according to the literature, expect neurological damage to occur in 1 to 6% of ERAH patients [31,34,42]. This is a limitation of this current study.

We found the expected incidence of complications, and, as seen in Table 4, with endoscopic harvesting, patients report an 18% incidence of sensory deficit. Though we have not quantified the severity of the sensory deficit, it would be expected, based on the literature, to cause half the morbidity of open harvest. This study also found it feasible to use a questionnaire in an upcoming randomized clinical trial comparing ORAH with ERAH focused on neurological deficits.

## 5. Conclusions

This study reports the risk of surgical complications immediately after ERAH. The patients reported an 18% occurrence of neurological deficits. Our questionnaire was feasible in a cohort of postoperative patients receiving radial artery harvest. We found that neurological deficits after radial artery harvest are both a relevant clinical problem and a significant occurrence, warranting a randomized clinical trial.

## Figures and Tables

**Table 1:** Demographic data.

*In general:*	*Mean*	*Median*	*Lowest Value*	*Highest Value*
Age (years)	60.8 (SD = 9.9)	61.3	39.0	85.5
Weight (kilograms—kg)	87.9 (SD = 15.5)	86.0	57.0	148.0
Height (meters—m)	1.76 (SD = 0.07)	1.76	1.56	2.00
Body mass index (kg per square m)	28.4 (SD = 4.4)	28.1	18.8	43.2
				
*Risk factors:*		*N* = 108	*Percent*	95% CI
Male		96	88.9%	90.5–98.4%
Hypertension		81	75.0%	66.1–82.2%
Hypercholesterolemia		91	84.3%	76.2–89.9%
Active smoker		37	34.3%	26.0–43.6%
Peripheral artery disease		12	11.1%	6.5–18.4%
Diabetes mellitus		32	29.6%	24.0–41.3%
Cerebral stroke preoperatively		5	4.6%	2.0–10.4%
Chronic obstructive pulmonary disease		7	6.5%	3.2–12.8%
Peripheral neuropathy		5	4.6%	2.0–10.4%
Nephropathy preoperative		4	3.7%	1.4–9.1%
Dialysis preoperative		0	0.0%	0.0–3.4%
Hereditary disposition		52	48.1%	39.0–57.3%
*Admission type:*		*N* = 108	*Percent*	95% CI
Elective		75	69.4%	59.7–77.7%
Subacute		33	30.6%	22.3–40.3%
*Coronary disease scope:*		*N* = 108	*Percent*	95% CI
Two-vessel disease		36	33.3%	24.7–43.1%
Three-vessel disease		72	66.7%	56.9–75.3%
Left main stem stenosis (LMS)		76	70.4%	60.7–78.6%
Non-LMS		32	29.6%	21.4–39.3%
	*Mean*	*Median*	*Lowest value*	*Highest value*
Left ventricle ejection fraction (ratio)	0.48 (SD = 0.11)	0.50	0.20	0.60
*General operative risk (n = 107):*	*Mean*	*Median*	*Lowest value*	*Highest value*
EuroSCORE II (percentage)	1.52% (SD = 1.20%)	1.14%	0.50%	6.42%

Abbreviations used: confidence interval (CI), kilogram (kg), left main stem stenosis (LMS), meter (m), Standard Deviation (SD).

**Table 2:** Perioperative data.

	n	Percent
*Procedure type (n = 107):*		
CABG	95	88.0%
OPCAB	3	2.8%
CABG + AVR	4	3.7%
CABG + Ablation	5	4.6%
Other	1	0.9%
*Number of distal anastomoses (n = 107):*		
0	1	0.9%
1	0	0.0%
2	17	15.9%
3	55	51.4%
4	27	25.2%
5	4	3.7%
6	2	1.9%
*Number of proximal anastomoses (n = 105):*		
0	3	2.9%
1	88	83.8%
2	16	15.2%
3	1	1.0%
*Use of conduits:*		
LIMA	100	92.6%
Both internal mammarian arteries	7	6.5%
No internal mammarian arteries	1	0.9%
RA-LIMA	81	75.0%
RA-RIMA	9	8.3%
RA-proximal aortic anastomosis	14	13.0%
RA not used	4	3.7%
Single vein graft	16	14.8%
Vein graft not used	92	85.2%

Abbreviations used: aortic valve replacement (AVR), coronary artery bypass grafting (CABG), left internal mammarian artery (LIMA), off-pump coronary artery bypass grafting (OPCAB), radial artery (RA), radial artery to left internal mammarian composite grafting (RA-LIMA), radial artery to right internal mammarian composite grafting (RA-RIMA).

**Table 3:** Postoperative complications.

	n	Percent	95% CI
*Post procedure general complications (n = 107):*			
Reoperation thorax due to bleeding	5	4.7%	1.8–11.1%
Reoperation due to pericardial tamponade	4	3.7%	1.2–9.8%
Postoperative coronary angiography	2	1.9%	0.3–7.3%
Reoperation due to graft failure	4	3.7%	1.2–9.8%
Postoperative atrial fibrillation	25	23.4%	16.0–32.8%
Postoperative ventricular tachycardia	2	1.9%	0.3–7.3%
Postoperative pneumothorax	4	3.7%	1.2–9.8%
Postoperative pneumonia	7	6.5%	2.9–13.4%
Mediastinitis	4	3.7%	1.2–9.8%
Postoperative sepsis	1	0.9%	0.0–5.8%
Acute kidney damage	4	3.7%	1.2–9.8%
Dialysis	1	0.9%	0.0–5.7%
Cerebral stroke	3	2.8%	0.7–8.6%
Mortality within 30 days	2	1.9%	0.3–7.3%
*Post procedure arm complications (n = 108):*			
Conversion ERAH to ORAH	0	0.0%	0.1–4.3%
Reoperation of the arm	0	0.0%	0.1–4.3%
Hematoma in the arm	0	0.0%	0.1–4.3%
Compartment in the arm	0	0.0%	0.1–4.3%
Ischemia in the arm	0	0.0%	0.1–4.3%
Neurological sensory damage in the arm	0	0.0%	0.1–4.3%
Neurological motor damage in the arm	0	0.0%	0.1–4.3%
Need for surgical drain in the arm	0	0.0%	0.1–4.3%
Infection in the arm	3	2.8%	0.7–8.5%
Wound dehiscence	0	0.0%	0.1–4.3%

Abbreviations used: confidence interval (CI), endoscopic radial artery harvest (ERAH), open radial artery harvest (ORAH).

**Table 4:** Interview responses ^1.^

	n	Percent	95% CI
*Post procedure infectious complications in the arm (n = 100):*			
**q3:** Treated for infection	7	7.0%	3.1–14.4%
**q3a:** Treated by general practitioner	7	100.0%	56.1–98.7%
**q3b:** Re-operation due to infection	0	0.0%	1.3–43.9%
**q3c:** Use of antibiotics	7	100.0%	56.1–98.7%
**q3d:** Days of antibiotics			
Mean = 8.7 Median = 10 Low = 5 High = 13			
**q3e:** Currently healed	7	100.0%	56.1–98.7%
*Post procedure hematoma formation in the arm (n = 100):*			
**q4:** Hematoma formation	2	2.0%	0.3–7.7%
**q4a:** Symptomatic	0	0.0%	4.9–80.2%
**q4b:** Influence current daily function	0	0.0%	4.9–80.2%
**q4c:** Re-operation due to hematoma	0	0.0%	4.9–80.2%
*Post procedure sensibility deficit in the arm (n = 100):*			
**q5:** Sensibility deficits	18	18.0%	11.3–27.2%
**q5a:** Current symptoms	12	12.0%	6.6–20.4%
**q5b:** Location of sensibility deficit			
1. finger = 44.4% 2. finger = 0.0% 3. finger = 5.6% 4. finger = 0.0% 5. finger = 5.6% palm = 0.0% dorsal = 5.6% Other = 38.9%			
**q5c:** Description of sensibility deficit			
Decrease = 22.2% Ceased = 5.6% Prickly = 50.0% Sleeping = 16.7%			
*Post procedure pain in the hand (n = 100):*			
**q6:** Hand pain	8	8.0%	3.8–15.6%
**q6a:** Current hand pain	7	7.0%	3.1–14.4%
**q6b:** Location of the pain			
1. finger = 25.0% 2. finger = 0.0% 3. finger = 12.5% 4. finger = 0.0% 5. finger = 0.0% palm = 0.0% dorsal = 0.0% Other = 62.5%			
**q6c:** Description of the pain			
Cutting = 37.5% Burning = 12.5% Prickly = 0.0% Other = 50.0%			
*Post procedure power reduction in the hand (n = 100):*			
**q7:** Power reduction	4	4.0%	1.3–10.5%
**q7a:** Current power reduction	2	2.0%	0.3–7.7%
**q7b:** Location of power reduction			
1. finger = 0.0% 2. finger = 0.0% 3. finger = 0.5% 4. finger = 0.0% 5. finger = 0.0% Other = 100.0%			
**q7c:** Degree of the power reduction			
No movement = 0.0% Some movement = 50.0% Non-significant = 25.0% Other = 25.0%			
**q8:** Current reduced function due to neurology	2	2.0%	0.3–7.7%
*Tendency to cold or pale fingers (n = 100):*			
**q9:** Cold or pale fingers	9	9.0%	4.5–16.8%
**q9a:** Cold fingers	8	8.0%	3.8–15.6%
**q9b:** Location of cold/pale fingers	9	9.0%	4.5–16.8%
1. finger = 2.2% 2. finger = 1.1% 3. finger = 0.0% 4. finger = 0.0% 5. finger = 0.0% Other= 6.6%			
**q9c:** Constant or provoked	7	7.0%	3.1–14.4%
Constant = 0.0% Cold = 7.7% Vibration = 0.0% Other = 0.0%			
**q10:** Current reduced function	0	0.0%	0.9–4.6%
*In general (n = 100):*			
**q11:** Postoperative angina	7	7.0%	3.1–14.4%
**q11a:** Postoperative myocardial infarction	0	0.0%	0.1–4.6%
**q11b:** Use of nitroglycerin	3	3.0%	0.8–9.5%
**q11c:** Postoperative coronary angiography	4	4.0%	1.3–10.5%
**q11d:** Postoperative percutaneous intervention	1	1.0%	0.1–6.2%
**q11e:** Renewed bypass surgery	0	0.0%	0.9–4.6%
**q12:** Satisfaction (0 to 10) Mean = 9.7 Median = 10 Minimum = 6 Maximum = 10			

^1^ Shortened text but full list of questions can be seen in Appendix B. Abbreviation used: confidence interval (CI).

## Data Availability

The original contributions presented in this study are included in the article. Further inquiries can be directed to the corresponding author.

## Abbreviations

The following abbreviations are used in this manuscript:

AVR	Aortic Valve Replacement
BMI	Body Mass Index
CABG	Coronary Artery Bypass Grafting
CI	Confidence Interval
ECC	Extra Corporal Circulation
ERAH	Endoscopic Radial Artery Harvest
LIMA	Left Internal Mammarian Artery
MVR	Mitral Valve Repair/Replacement
NEO	Neurological complications in Endoscopic versus Open radial artery harvest
OPCAB	Off-Pump Coronary Artery Bypass
ORAH	Open Radial Artery Harvest
PATS	Patient Advocate Tracking System
RA	Radial Artery
RAPCO	Radial Artery Patency and Clinical Outcomes Trial
RIMA	Right Internal Mammarian Artery
SD	Standard Deviation
SVG	Saphenous Vein Graft

## Appendix A

### List of Clinical Data Collected

Demographic data: Weight, height, sex (m/f), dominant arm (r/L).

Risk factors: hypertension (y/n), hypercholesterolemia (y/n), smoking (y/n), peripheral arterial disease (y/n), diabetes mellitus (n/type1/type2/type2 + insulin), apoplexy cerebri (y/n), chronic obstructive pulmonary disease (y/n), peripheral neuropathy (y/n), nephropathy (y/n), dialysis preoperative (y/n), genetical disposition (y/n).

Preoperative data: modified Allen’s test (n/y-usable/y-not usable), case priority (elective/under admission/acute), no. vessel disease (1/2/3), left main stem stenosis (percentage), left ventricular ejection fraction (percentage), myocardial infarction inside 90 days (y/n), no. myocardial infarction before (number), preoperative inotropic (y/n), preoperative infusion of nitro-glycerine (y/n), EuroSCORE I (percentage), EuroSCORE II (percentage).

Perioperative data: procedure (CABG/OPCAB/CABG + AVR/CABG + Maze/CABG + MVR/Other), peripheral anastomosis (number), proximal anastomosis (number), internal mammarian artery used (n/left/right), radial artery used and if so in which position (n/LIMA/aorta), saphenous vein anastomosis (number).

Postoperative data: reoperation for bleeding (y/n), re-angiography after surgery (y/n), re-operation for graft-failure (y/n), atrial fibrillation (y/n), ventricular fibrillation/tachycardia (y/n), pneumothorax (y/n), pneumonia (y/n), mediastinitis (y/n), sepsis (y/n), acute nephropathic injury (y/n), dialysis (y/n), apoplexy cerebri (y/n), intensive care unit stay (no. days), admittance (no. days), creatinine kinase MB (number), mortality inside 30 days (y/n), date of mortality (date).

Complications to radial artery harvesting according to patient file: conversion to open radial artery harvesting (y/n), re-operation of the arm (y/n), hematoma formation (y/n), compartment in donor arm (y/n), ischemic symptoms of the hand or arm (y/n), neurological damage—sensory (y/n), neurological damage motoric (y/n), radial artery not usable (y/n), drain placed in arm during operation (y/n), infection (n/antibiotics/culture), dehiscence of donor arm (y/n).

## Appendix B

### List of Interview Questions

- Is it alright to ask you some questions? (y/n) ***q1***.
- From which hand did you get an artery removed? (right/left) ***q2a***.
- Which hand do you use to write? (right/left) ***q2b***.
- Were you satisfied with your hospitalization considering the surgery on the arm? (y/n) ***q2c***.
- Infection: Did you get treatment for infection of your wound in the arm? (y/n) ***q3***; Who treated you? (department/general practitioner) ***q3a***; Did you need re-exploration? (y/n) ***q3B***; Did you get antibiotics? (y/n—and if “y” which kind?) ***q3c***; How long did you get antibiotics? (days) ***q3d***; Has it now healed satisfactory? (y/n) ***q3e***.
- Bleeding: Did you after the operation get at hematoma in your arm? (y/n) ***q4***; If “y”—Did the hematoma give you symptoms? (y/n) ***q4a***—if “y” which symptoms?; Does the hematoma today influence your daily function? (y/n) ***q4b***; Did you get re-operated during your in-hospital stay? (y/n) ***q4c***.
- Sensibility: Did you after the operation have any new deficits in sensibility in the arm? (y/n) ***q5***; If “y”—Do you this day have any deficits in sensibility in the arm? (y/n—and if “n” when did your sensibility normalize? (date) ***q5a***; Where did you have/do you have decreased sensibility? (1. Finger/2. Finger/3. Finger/4. Finger/5. Finger/palm/dorsal hand/other) ***q5b***; How would you describe the change in sensibility? (decrease/ceased/prickly or sleeping sensation) ***q5c***.
- Pain: Did you after the operation have any new pain in the hand? (y/n) ***q6***; If “y”—Do you still have pain in the hand?—if “n” when did the pain go away? (date) ***q6a***; Where was the pain situated? (1. Finger/2. Finger/3. Finger/4. Finger/5. Finger/palm/dorsal hand/other) ***q6b***; How do you best describe the pain? (cutting/burning/prickly/other) ***q6c***.
- Power: Did you after the operation experience power reduction in the hand? (y/n) ***q7***; if “y”—Do you today still have power reduction in the hand? (y/n) ***q7a***—if “n” when did the power get back to normal? (date); Where was the power reduction localized? (1. Finger/2. Finger/3. Finger/4. Finger/5. Finger/other) ***q7b***; How significant was the power reduction? (No movement/some movement/non-significant/other) ***q7c***; Do you in any way today have a reduced function in your hand due to neurological damage? ***q8***.
- Blood supply: Did you after the operation have tendency to cold or pale fingers? (y/n) ***q9***; If “y”—Is it cold or pale fingers? (cold/pale) ***q9a***; Which fingers have a tendency to become cold or pale? (1. Finger/2. Finger/3. Finger/4. Finger/5. Finger/other) ***q9b***; Are the symptoms constant or can they be provoked by anything specific? (constant/cold/vibration/other) ***q9c***; Do you in any way have a decreased hand function as a result of declined blood supply? (y/n) ***q10***.
- Final questions: Have you since the operation had angina pectoris? (y/n) ***q11*** and if “y” (1) Have you been admitted to hospital with acute myocardial infarction? (y/n) ***q11a***; (2) Do you take nitroglycerin against chest pain? (y/n) ***q11b***; (3) Have you had a new coronary angiogram? (y/n); (4) Did you get a percutaneous balloon or stent intervention? (y/n) ***q11d*** and (5) Have you been referred for a new bypass procedure? (y/n) ***q11e***.
- How satisfied on a scale from 0 to 10, have you been with the use of endoscopic technique to remove the artery from your arm? (0/1…/10) ***q12***.
- If you yourself had to pay the price of 700 € in order to use endoscopic technique, would you consider it worth the price? (y/n/do not know) ***q13***.
- Do you think the technique has made your postoperative recovery easier and/or shorter? (y/n/do not know) ***q14***.
- Would you be willing to come to Rigshospitalet in order to get at CT-scan if a project about this technique was established? (y/n/do not know) ***q15***.
- Are we allowed to contact you at a later point in time with more questions about your operation? (y/n) ***q16***.
- Do we have your verbal informed consent under consideration of duty of confidentiality to use your operative data in a scientific article about the endoscopic technique? (y/n) ***q17***.
